# Effect of hnRNPA2/B1 on the proliferation and apoptosis of glioma U251 cells via the regulation of AKT and STAT3 pathways

**DOI:** 10.1042/BSR20190318

**Published:** 2020-07-09

**Authors:** Decheng Yin, Chengxiang Kong, Muhu Chen

**Affiliations:** 1 Department of Emergency Medicine, The Affiliated Hospital of Southwest Medical University, Luzhou 646100, China; 2 Department of PICU, Baoan Maternal and Child Health Hospital, Jinan University, Shenzhen 518106, China

**Keywords:** apoptosis, glioma, hnRNPA2/B1, proliferation

## Abstract

Glioma is the most common malignant tumor in the human central nervous system. Although heterogeneous nuclear ribonucleoprotein A2/B1 (hnRNPA2/B1) was previously presumed to be a tumor-promoting gene, the relationship between hnRNPA2/B1 and glioma is unclear. Targeting hnRNPA2/B1 interference in glioma cells can significantly inhibit proliferation and increase apoptosis of human glioma cells *in vitro*. In a tumor xenograft model, knockdown of hnRNPA2/B1 suppressed tumor growth in glioma cells *in vivo*. In terms of a mechanism, the knockdown of hnRNPA2/B1 led to inactivation of the AKT and STAT3 signaling pathways, which ultimately reduced the expression of B-cell lymphoma-2 (Bcl-2), CyclinD1 and proliferating cell nuclear antigen (PCNA). Collectively, these data suggest that the inhibition of hnRNPA2/B1 can reduce the growth of gliomas through STAT3 and AKT signaling pathways, and this inhibition is expected to be a therapeutic target for gliomas.

## Introduction

Gliomas originate from neuroepithelial tissue and represent the most common primary malignant tumor in the adult central nervous system. The incidence rate of glioma is approximately 7.2 per 100000 people per year [1,2]. The median survival of patients with glioma is only 12–15 months [3]. Maximal resection of the tumor, followed by further radiotherapy and chemotherapy, is the current comprehensive treatment standard for patients with glioma [4]. Although comprehensive treatment is now widely used, the prognosis and results are not satisfactory [5]. The invasive growth pattern of glioma makes it difficult to completely remove the tumor, which is the main cause of the high mortality of the disease [6]. Because of the failure of conventional strategies, understanding the molecular mechanisms of gliomas will help to accurately deliver tumor-killing drugs to advance the treatment of the disease.

Heterogeneous nuclear ribonucleoprotein A2/B1 (hnRNPA2/B1) is an important member of the hnRNP family, with closely related sequences and a conserved modular structure [7]. This family can usually affect alternative splicing by blocking the SR protein and, in part, by identifying exon splicing silencing elements [8]. It has been reported that these proteins also have additional functions in the splicing process, such as mRNA transport, cytoplasmic RNA virus replication and transcription [7]. hnRNPA2/B1 is related in carcinogenesis through its interaction with other proteins [9,10], including pancreatic cancer, breast cancer, gastrointestinal tumors and nervous system tumors [11]. hnRNPA2/B1 has been reported to mediate the pathogenesis of gliomas via the classic STAT3 and AKT-related signaling pathways [12,13]. hnRNPA2/B1 knockout reduces cell viability, migration and invasion and decreases P-STAT3 and MMP-2 in glioblastoma cells [14]. There is evidence that β-asarone induced apoptosis and cell cycle arrest of glioma cells inactivates AKT signaling regulate Bcl-xS, Bcl-xL p21, p27, Cdc25A, cyclin D, cyclin E and CDK2 [15]. Under cancer conditions, hnRNPA2/B1 and its downstream mediators PI3K/Akt/mTOR and JAK/STAT3 play important roles in inhibiting the release of apoptotic factors and cell proliferation [16,17]. Recently, an increasing number of studies have reported that the AKT and STAT3 signaling pathways regulate B-cell lymphoma-2 (Bcl-2), proliferating cell nuclear antigen (PCNA), CyclinD1 and other factors and have obvious biological effects on tumors such as oral cancer, renal cancer and follicular lymphoma [18–20]. The above studies indicate the important role of hnRNPA2/B1 in carcinogenesis, invasion and metastasis. However, the precise molecular mechanism of hnRNPA2/B1 in the glioma development have not been comprehensively investigated.

In this experiment, we analyzed the function of hnRNPA2/B1 via RNA interference, which is a relatively common technology scientists study gene function. First, knockdown hnRNPA2/B1 in glioma cells. Then, the effect of this gene on the proliferation and apoptosis of glioma cells was investigated. Finally, the related mechanisms of the function of hnRNPA2/B1 are addressed.

## Materials and methods

### Antibodies and other materials

All chemicals were purchased from Sigma Chemical Co. (St. Louis, MO) unless otherwise specified. The primary antibodies for t-AKT, p-AKT, t-STAT3, p-STAT3, Bcl-2 were obtained from Cell Signaling Technology (CST Inc., U.S.A.). The primary antibodies for PCNA, CyclinD1, hnRNPA2/B1, and β-actin as well as all the secondary antibodies were obtained from Santa Cruz Biotechnology (Santa Cruz, CA, U.S.A.). The recombinant plasmid for the human hnRNPA2/B1 gene transcription sequence with green fluorescent protein (GFP) was obtained from Karma Biotechnology (Wuhan, China). The transfection reagent MegaTran 1.0 was obtained from OriGene (CA, U.S.A.). TRIzol reagent, the Reverse Transcription Kit and RT-PCR Primers were obtained from TaKaRa (Kusatsu, Japan), and the Total Cell Protein Extraction Kit and BCA Protein Concentration Assay Kit were obtained from Beyotime Biotechnology (Shanghai, China).

### Cell culture

The U251 glioma cell line was obtained from the Shanghai Institute of Biological Sciences Cell Bank. The cells were maintained in DMEM medium containing 10% fetal bovine serum. Glioma cells (37°C, 5% CO_2_, 95% humidity) were cultured and passaged until 80% were adherent to growth.

### Real-time PCR

The total RNA was extracted from U251 glioma cells using an RNAiso Plus (TaKaRa) Kit. The RNA concentration of these samples was measured using a spectrophotometer, and then the RNA samples were reverse transcribed into cDNA using a Primescript RT kit (TaKaRa) followed by the protocol. Next, 10 μl of the RNA samples was applied to the reaction system, with the following amplification conditions: predenaturation at 95°C for 3 min, 95°C for 10 s and 56°C for 30 s, for a total of 40 cycles. The following primer sequences were used: hnRNPA2/B1-F: 5′-AGCGACTGAGTCCGCGATGGA-3′; hnRNPA2/B1-R: 5′-GCAGGATCCCTCA TTACCACACAGT-3′, with a total of 241 bp. Cellular hnRNPA2/B1 and β-actin mRNA levels were detected using the SYBR Green Real-time PCR kit and a fluorescent real-time PCR instrument (Bio-Rad).

### RNA interference

The hnRNPA2/B1 mRNA sequence (NM-031243) in GenBank was obtained to construct a recombinant plasmid for the transcribed region sequence. Sequence information justice chain: 5′-CCGGAGAAGCTGTTTGTTGGCGGAACTCGAGT TCCGCCAACAAACAGCTTCTTTTTTG-3′; antisense strand: 5′-AATTCAAA AA AGAAGCTGTTTGTTGGCGGAACTCGAGTTCCGCCAACAAACAGCTTCT -3′, 1054 bp, and the pLK0.1/control empty plasmid was used as a control.

### Plasmid transfection

Transfection was performed according to the instructions for the use of the reagents. After transfection, the transfection efficiency was observed by fluorescence microscopy. After transfection for 48 h, DMEM containing G418 (800 μg/ml) and 10% newborn calf serum was replaced for resistance screening for 2 weeks. After cloning was selected by fluoroscopy, the medium containing 400 μg/ml G418 and 10% newborn calf serum was used for maintenance and observed under a fluorescence microscope.

### Western blot analysis

The cells were collected for total protein extraction, and the protein concentration measurement kit was used to measure the protein concentration. A total of 40 μg of total protein was taken and electrophoresed on 10% SDS/PAGE, and then the protein was electrotransferred to a PVDF membrane and blocked with PBST (containing 5% skim milk powder) for 1 h. After that, the membranes were incubated with primary antibodies overnight at 4°C, including hnRNPA2/B1 (1:1000), t-AKT (1:1000), p-AKT (1:1500), t-STAT3 (1:1000), p-STAT3 (1:1000), β-actin (1:1000), PCNA (1:2000), Bcl2 (1:800) and CyclinD1 (1:1000). The PVDF membrane was washed three times with TBST for 10 min each, and, the membrane was incubated with HRP-labeled secondary antibody (diluted 1:5000) at 37°C for 2 h; then, the membrane was washed three times with TBST for 10 min each, followed by BeyoECL Plus chemiluminescence Method development. The Bio-Rad gel imaging system collected images, and the strips were quantified using Quantity One software. β-actin was used as an internal reference.

### Flow cytometric assay to detect apoptosis

The cells from each group were collected, washed with 1× buffer, and resuspended, and then the precipitate was collected. Next, the cells were mixed with 300 μl of 1× binding buffer, and 5 μl of Annexin V-FITC was added for 15 min in the dark. Then, 5 μl of iodide was added. The apoptosis rate was measured by flow cytometry using propidium dye solution and 200 μl of 1× binding buffer. The experiment was repeated five times.

### Flow cytometric assay to detect the cell cycle

Each group of cells was collected and fixed in 75% ethanol solution. Then, 200 μl of 1 mg/ml RNase A solution was added and the solution was incubated at 37°C for 30 min. Next, 800 μl of Propidium Iodide staining solution was added, and the solution was mixed thoroughly and allowed to rest at room temperature. The cells were stained for 30 min in the dark at 4°C. Finally, the cell cycle was detected with a flow cytometer. The experiment was repeated three times.

### MTT colorimetric assay for cell proliferation

The cells were plated, and 200 μl of each suspension (approximately 6 × 10^3^ cells) was seeded in a 96-well plate. After incubation for 24, 48 and 72 h, 20 μl of MTT working solution was added for 4 h; then, the solution was mixed with 120 μl DMSO and shaken for 10 min, and the absorbance value (D492) was measured by enzyme spectroscopy. The experiment was repeated three times.

### Plate cell colony formation assay for cell proliferation

A cell suspension was prepared, and a density of 50 cells per well was uniformly inoculated into a six-well plate containing 2.5 ml of a 37°C culture solution. Macroscopic clones were visually observed for more than 10 days in static culture; the supernatant was discarded, and the clones were carefully washed with PBS twice. The clones were fixed with 5 ml of methanol for 15 min. Then, for the fixative solution, an appropriate amount of Giemsa staining solution was added for 30 min, and the staining solution was slowly washed off with running water and allowed to dry at room temperature. A transparent film with a small grid on the surface was placed on the clones in order to carefully count the clones with the naked eye; then, the clone formation rate was calculated.

### Xenograft tumor model

Male nude mice (4 weeks old) used in the present study were provided by the Experimental Animal Center of Southwest Medical University. *In vivo* experiments in our study were performed in the Experimental Animal Center of Southwest Medical University. All animal studies were approved by the Ethics Committee of Southwest Medical University (20180817002). The glioma cells were resuspended in DMEM at a density of approximately 2 × 10^6^, and each 50 µl sample of cells was injected subcutaneously into the nude mice. Subcutaneous tumor volume was recorded in nude mice 7, 14, 21 and 28 days after inoculation. The tumor size was estimated as (length ×width^2^)/2. On the 28th day, mice were killed by ketamine anesthesia intraperitoneally (100 mg/kg body weight) and the xenografts were harvested for further detection.

### Statistical analysis

Statistical differences between groups were analyzed by *t* tests or one-way ANOVA using SPSS 19.0. *P*<0.05 was considered to be a statistically significant difference. All data are expressed as the mean ± standard deviation (SD).

## Results

### Construction of a glioma-stabilized cell line by knocking down hnRNPA2/B1 transfection

**Figure 1:**
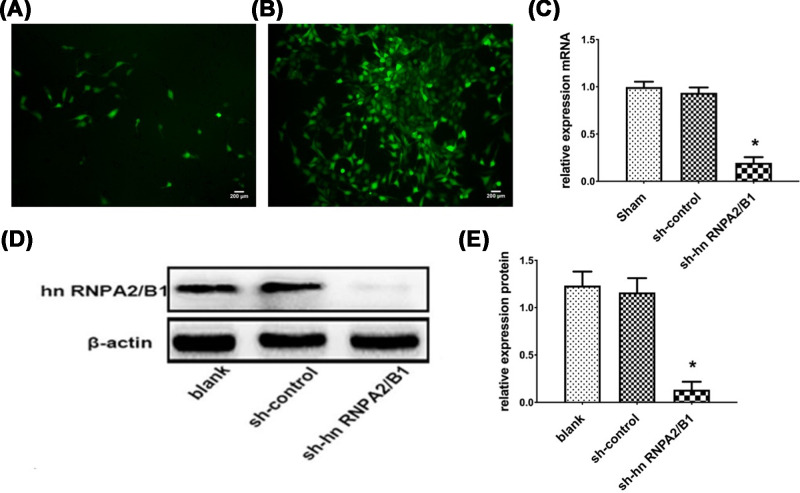
Successfully constructed glioma stable cell line that knocks down hnRNPA2/B1 (**A**) hnRNPA2/B1 after 24 h of transfection. (**B**) hnRNPA2/B1 transfected for 2 weeks and screened by G418 (×200). (**C**) RT-qPCR analysis of hnRNPA2/B1 mRNA expression levels in the transfected group (*P=0.0142, <0.05 vs control, analyzed by *t* test). (**D,E**) Western blot analysis of hnRNPA2/B1 protein expression levels in the transfected group, blank group (cells with only transfection reagent), sh-control group (transfected with control shRNA); and sh-hnRNPA2/B1 group (transfected with sh-hnRNPA2/B1 vectors). Data were based on at least three independent experiments and are shown as the mean ± SD (*P=0.0188, <0.05 vs control, analyzed by *t* test).

After 24 h of transfection, green fluorescence was observed (Figure 1A). After 48 h, the complete medium containing G418 (600 μg/ml) was replaced for resistance cell screening. After 2 weeks, the monoclonal cancer cell line was successfully established (Figure 1B). The results of RT-qPCR showed that the hnRNPA2/B1 mRNA in the knockdown group was significantly lower than that in the blank group and the control group, and the expression inhibition rate was 0.80 ± 0.04 (Figure 1C). The hnRNPA2/B1 protein was down-regulated 8.6-fold in the knockdown group compared with that in the control group (Figure 1D,E).

### Inhibition of hnRNPA2/B1 promotes apoptosis of glioma cells

**Figure 2:**
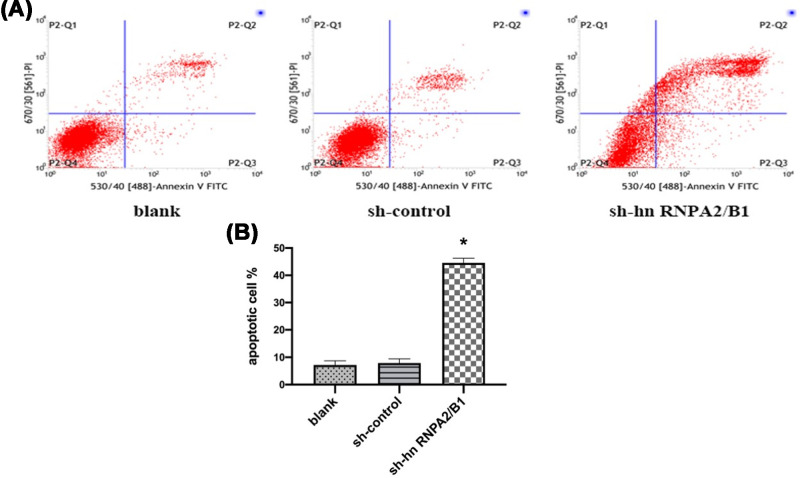
Inhibition of hnRNPA2/B1 promotes apoptosis of U251 cells (**A**) Cell apoptosis was analyzed by Annexin V/PI staining. (**B**) Analysis of the percentage of apoptotic tumor cells. Data were based on at least three independent experiments and are shown as the mean ± SD (*P=0.0070, <0.01 vs control, analyzed by *t* test).

We developed hnRNPA2/B1 stable knockdown in U251 glioma cells. To understand the functional mechanism of growth inhibition, Annexin V/PI staining assay was performed to detect apoptosis in sh-hnRNPA2/B1 cells. Total apoptotic rate = early apoptotic rate (P2-Q3) + late apoptotic rate (P2-Q2). The total apoptosis rate of the sh-hnRNPA2/B1 group was significantly increased (41.78 ± 1.76)% compared with that of the control group, and late apoptosis was mainly increased (Figure 2). The difference was statistically significant.

### Inhibition of hnRNPA2/B1 attenuates glioma cell proliferation

**Figure 3:**
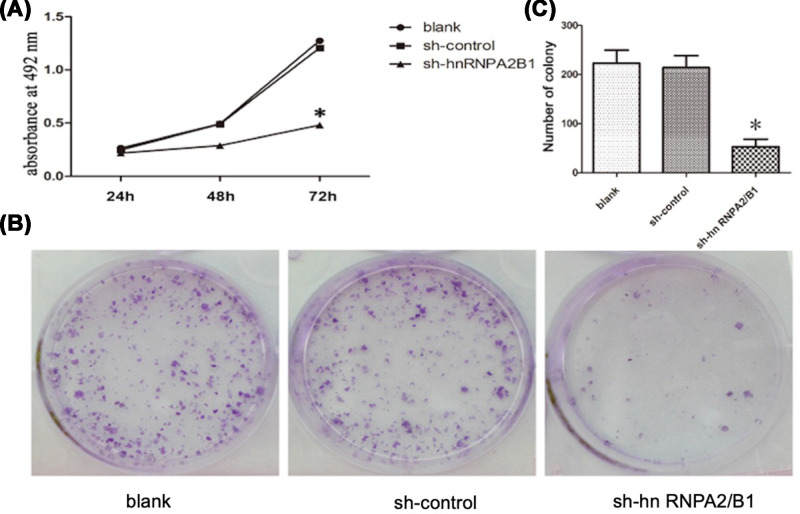
Inhibition of hnRNPA2/B1 attenuates the proliferation and colony forming ability of U251 cells (**A**) Cell proliferation was detected in U251 cells by MTT assay (*P=0.0137, <0.05 vs control, analyzed by *t* test). (**B**) Detection of clonality in U251 cells by plate colony formation assay. (**C**) Analysis of the clone number of tumor cells. Data were based on at least three independent experiments and are shown as the mean ± SD (*P=0.0088, <0.01 vs control, analyzed by *t* test).

The cell proliferation rate of the sh-hnRNPA2/B1 group significantly decreased by (2.89 ± 0.19)%, (19.97 ± 1.16)% and (72.57 ± 3.42)%, 24, 48 and 72 h after transfection, respectively, compared with that of the control group (Figure 3A). The number of colonies containing over 50 cells was counted and recorded. Clonal formation rate = (number of clones/number of cells inoculated) × 100%. The number of clones in the sh-hnRNPA2/B1 group was 47.83 ± 5.59. The number of cell clones was significantly reduced and the cell proliferation ability was significantly inhibited in the sh-hnRNPA2/B1 group compared with those in the control group (Figure 3B,C). The difference was statistically significant.

### Inhibition of hnRNPA2/B1 prolongs the cell cycle, which appears to be arrested in S-phase

**Figure 4:**
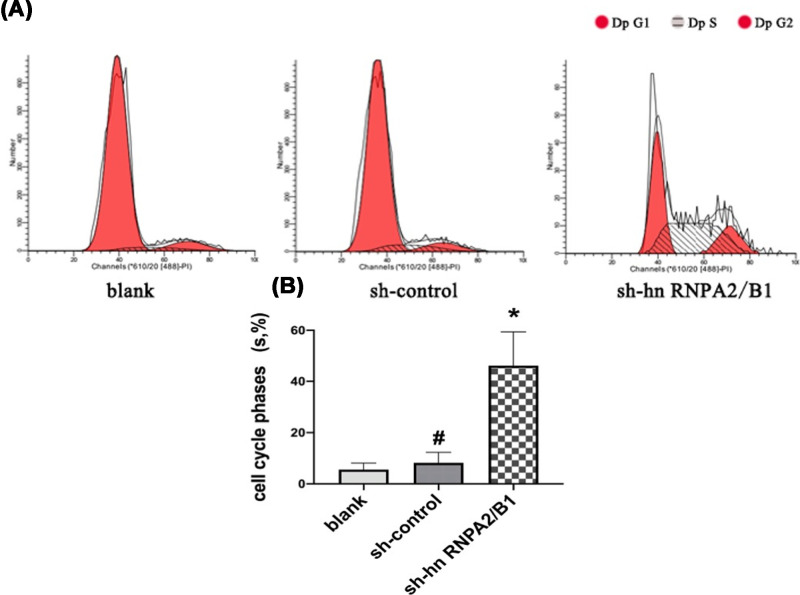
Knockdown of hnRNPA2/B1 inhibited the proliferation of glioma cells (**A**) Cell cycle distributions were tested in U251 cells by flow cytometry. (**B**) Analysis of the percentage of S-phase in the whole cell cycle. Data were based on at least three independent experiments and are shown as the mean ± SD (*P=0.0258, <0.05 vs control, analyzed by *t* test),^ #^no statistical difference.

The S-phase of the blank group was (4.53 ± 1.31)%, compared with that of the sh-control group (7.72 ± 2.54)%, the difference was not significant (*P*=0.1037, >0.05). The S-phase of the sh-hnRNPA2/B1 group was (44.73 ± 4.32)%; compared with that of the sh-control group, there was a significant S-phase prolongation, indicating that the DNA replication rate was significantly slower (Figure 4).

### Knockdown of hnRNPA2/B1 reduces AKT and STAT3 signaling pathway phosphorylation in glioma cells

**Figure 5:**
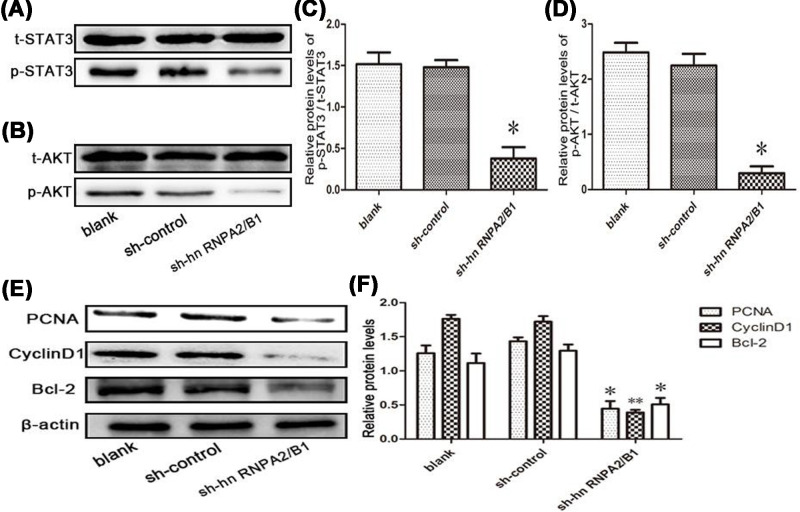
Knockdown of hnRNPA2/B1 inhibits AKT and STAT3 signaling in glioma cells (**A–D**) Western blot analysis of AKT and STAT3 phosphorylation levels (*PC = 0.0317, <0.05; *PD = 0.0142, <0.05 vs control, analyzed by *t* test). (**E,F**) Analysis of PCNA, Cyclin D1 and Bcl-2 expression by knockdown of hnRNPA2/B1 in U251 cells. Data were based on at least three independent experiments and are shown as the mean ± SD (*P=0.0209, <0.05; **P=0.0082, <0.01 vs control, analyzed by *t* test).

Due to the previously observed phenomena, we further explored the molecular mechanism by which the knockdown of hnRNPA2/B1 attenuates glioma proliferation and enhances apoptosis. We next explored the effects of the knockdown of hnRNPA2/B1 on these two signaling pathways in glioma cells and their regulation of cell growth and apoptosis. We found that the knockdown of hnRNPA2/B1 in glioma cells strongly inhibited AKT and STAT3 phosphorylation (Figure 5A–D). The protein expression of phospho-STAT3 and phospho-AKT was significantly decreased in the hnRNPA2/B1 group compared with that in the control group. Knockdown of hnRNPA2/B1 inhibited the expression of PCNA, CyclinD1, and Bcl2 (Figure 5E,F). These data may explain the role of hnRNPA2/B1 in promoting cell proliferation and inhibiting apoptosis.

### hnRNPA2/B1 promotes tumor formation in a xenograft model

**Figure 6:**
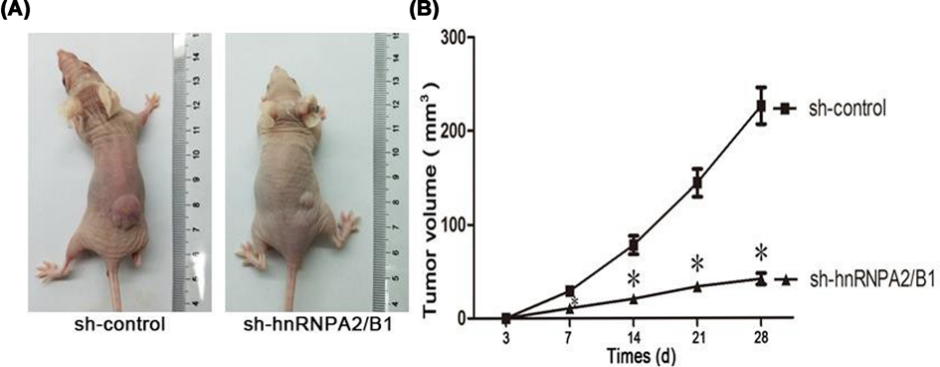
hnRNPA2/B1 promotes tumor formation in a xenograft model (**A**) A typical image of a subcutaneous tumor xenograft in nude mice after inoculation with U251 cells silenced with sh-hnRNPA2/B1 or its control plasmid. (**B**) Time course analysis of tumor growth after injection. Data were based on at least three independent experiments and are shown as the mean ± SD (*P<0.05 vs control, analyzed by one-way ANOVA).

To further verify the conclusion that hnRNPA2/B1 promotes tumor formation *in vitro* and whether the results are consistent with the *in vitro* results, we injected glioma cells into the body for observation. The effect of hnRNPA2/B1 on tumor growth was investigated using a xenograft model. Tumors gradually became apparent during the study period, and mice were killed after 28 days of transfection. The growth rate of glioma cells was reflected by measuring tumor volume. hnRNPA2/B1 silencing slowed tumor growth (Figure 6A,B). Therefore, this observation clearly demonstrated that hnRNPA2/B1 can significantly enhance the tumorigenicity of U251 cells *in vivo*.

## Discussion

Glioma is the most common primary tumor of the central nervous system. The treatment of glioma is currently based on surgery, combined with radiotherapy, chemotherapy, and other complementary methods of traditional Chinese medicine [21]. Even now, some gene therapy and individualization methods exist. While the course of treatment is well identified, the overall treatment effects are still unsatisfactory; thus, it is imperative to find effective treatment methods.

hnRNPA2/B1 is an important RNA connexin and is the main component of the nuclear heterogeneous nuclear riboprotein core complex [22]. hnRNPA2/B1 accounts for the highest proportion of the A/B subtypes. The two components are derived from the same gene and are located on human chromosome 7p15, which is approximately 11.39 kb long and covers 12 exons and 11 introns. The molecular weights of hnRNPA2 and hnRNPB1 are 36 and 38 kDa, respectively [23]. hnRNPB1 inserts 36 bases at the 5′ end of the hnRNPA2 gene sequence, thus, hnRNPB1 has 12 more amino acids than hnRNPA2 [24]. In the hnRNPA2/B1 complex, the ratio of A2 to B1 is approximately 3:1, but the ratios are different in different tissues. According to the high homology of the two components, scholars collectively call them hnRNPA2/B1 [25]. hnRNPA2/B1 has wide and low-level expression in normal tissues, but the expression in different organ tissues varies widely. This complex is involved in many key pathophysiological processes of cells, such as RNA splicing, mRNA nuclear-cytoplasmic transport, posttranscriptional regulation, pre-mRNA maturation and degradation, telomere and telomerase DNA sequence regulation, cell mitosis, maturation, differentiation and the regulation of apoptosis [26]. Many studies have found that hyperproliferation and invasion of tumor cells are closely related to biological behaviors and prognosis, such as tumor occurrence, invasion and metastasis [27–29]. hnRNPA2/B1 has been overexpressed in various tumor diseases, such as breast cancer, liver cancer, colorectal cancer, and pancreatic cancer. Overexpression of hnRNPA2/B1 in non-small cell lung cancer accelerates cell proliferation, while down-regulation of hnRNPA2/B1 enhances apoptosis in breast cancer cells [30,31]. In cervical cancer, the knockdown of hnRNPA2/B1 can inhibit cell proliferation, invasion and cell cycle arrest through the PI3K/AKT signaling pathway and can also induce apoptosis in cervical cancer cells [32]. Knockdown of the hnRNPA2B1 gene can significantly inhibit the proliferation of breast cancer cells, induce apoptosis of cancer cells, prolong the cell cycle and induce cell cycle arrest in S-phase. The effect of hnRNPA2B1 on the occurrence and development of breast cancer may be through the role of STAT3 and ERK1/2-related signaling pathways [33]. Our results are in accordance with these reports demonstrating that inhibition of hnRNPA2/B1 promoted apoptosis and attenuated glioma cell proliferation via arrested the cell cycle in S-phase of glioma cells. In hepatoma cells, hnRNPA2/B1 induces EMT and promotes the metastasis by activating the transcription factor SNAIL [34]. Here, we show that alternative splicing of an exon in the 5′ untranslated region of a gene termed TP53INP2 is a key event downstream of hnRNP A2 that is necessary for cells to invade the extracellular matrix. We report that although siRNA of hnRNP A2 had little influence on the ability of cells to migrate on plastic surfaces, the splicing regulator was clearly required for cells to move effectively on three-dimensional matrices and to invade into plugs of extracellular matrix proteins. We used exon-tiling microarrays to determine that hnRNP A2 controlled approximately six individual splicing events in a three-dimensional matrix-dependent fashion, one of which influenced invasive migration.

To understand the potential biological role of hnRNPA2/B1 in gliomas, we constructed a glioma cell line that knocked down hnRNPA2/B1. The effects of hnRNPA2/B1 on the proliferative potential of glioma cells were observed by MTT and plate cloning experiments. The results showed that knockdown of hnRNPA2/B1 inhibited the proliferation of U251 cells. Flow cytometry showed that apoptosis can be induced by knocking down hnRNPA2/B1, which may contribute to the treatment of gliomas. Our work also found that hnRNPA2/B1 knockdown induced cell cycle arrest in S-phase in glioma, which means that the cell cycle arrested during DNA replication. We hypothesize that RNPA2/B1 can stimulate tumor proliferation by accelerating DNA replication, and further study in-depth mechanism research is needed. In addition, the subcutaneous tumor model in nude mice showed that knockdown of hnRNPA2/B1 significantly reduced the growth of gliomas *in vivo*.

We analyzed whether hnRNPA2/B1 can influence the development of tumors by regulating the balance of glioma cell growth and apoptosis. Thus, hnRNPA2/B1 can mediate abnormal survival signals to drive tumorigenesis. It is known that the PI3K/AKT signaling pathway plays a role in various human cells and regulates cell cycle and energy metabolism as well as affects the physiological functions of various cells. In recent years, there have been an increasing number of reports on AKT in various tumors, and it has become increasingly obvious that AKT has a great influence on tumors. In tumor cells, AKT phosphorylates p-AKT under the action of PI3K, thereby acting, such as through the activating of downstream-related transcription factors, to regulate cell proliferation, apoptosis, autophagy, invasion etc [35]. Some scholars have reported that the expression of AKT is positively related to the malignant grade of glioma. When AKT is overexpressed, it will lead to the increased value-adding ability and invasiveness of glioma [36]. After measuring phosphorylated AKT, we found that hnRNPA2/B1 is a potent agonist of the PI3K/AKT pathway consistent with carcinogenic potential. This finding may mean that hnRNPA2/B1 is required for activation of this pathway in glioma cells. Another signaling pathway that has long been associated with apoptosis and survival of tumor cells [37], the STAT3 signaling pathway, which is activated by phosphorylation in glioma cells and has regulatory functions in terms of proliferation, angiogenesis, tumor migration, invasion [38]. However, there is currently no clear study on the association of STAT3 with hnRNPA2/B1. Based on our results, AKT phosphorylation was inhibited after silencing the hnRNPA2/B1 gene in glioma U251 cells. And silencing hnRNPA2/B1 inhibits STAT3 phosphorylation and decreases STAT3 activity; Therefore, we believe that the role of hnRNPA2/B1 in the development of glioma may be related to the AKT pathway and STAT3 pathway.

It has been reported in the literature that AKT and STAT3 have also been identified to be involved in the regulation of downstream factors, including PCNA [39], Bcl2 [40,41] and CyclinD1 [39,41], and to manage tumor proliferation and apoptosis. Since the knockdown of hnRNPA2/B1 was observed to reduce cell proliferation and since apoptosis was activated, we reasonably speculated that this was achieved by inhibiting the expression of PNCA, CyclinD1 and Bcl-2 and reducing the phosphorylation of AKT and STAT3. These findings suggest that hnRNPA2/B1 may stimulate tumor growth and inhibit cell apoptosis in glioma by promoting the activation of AKT and STAT3 signaling pathways.

Due to the difficulty in collecting surgical tumor specimens, the number of samples in studies is insufficient, which leads to a lack of correlation between the hnRNPA2/B1 gene and glioma in glioma specimens from surgical patients; these results are similar to those seen with other human tumor characteristics. Because related studies about hnRNPA2/B1 are still in the laboratory stage, there is still a long way to go for hnRNPA2/B1 gene-targeted therapies for tumor as well as clinical drug research; thus, our research is temporarily unable to use drugs to interfere with hnRNPA2/B1. However, based on the observations observed in our experiments and the subsequent in-depth exploration, we should be able to comprehensively grasp the role of hnRNPA2/B1 in the biological behaviors of glioma proliferation, invasion, metastasis and recurrence as well as the specific mechanism of the above effects. Based on these studies, it may be possible to treat glioma patients with hn RNPA2/B1 gene inhibitors in the future, which is expected to greatly improve the therapeutic effect of glioma and increase the long-term survival of patients. Therefore, the present study provides a new theoretical basis for the further study of biological behaviors of gliomas, suggests the development of new targets for glioma gene therapy for hnRNPA2/B1, and ultimately helps solve the clinical treatment concern for glioma.

## Abbreviations

Bcl-xL: B-cell lymphoma-extra large
Bcl-xS: B-cell lymphoma-extra small
Bcl-2: B-cell lymphoma-2
Cdc25A: cell division cycle 25A
CDK2: cyclin-dependent kinase 2
G418: geneticin
hnRNPA2/B1: heterogeneous nuclear ribonucleoprotein A2/B1
HRP: horseradish peroxidase
MMP-2: matrix metallo proteinase-2
MTT: 3-(4,5-dimethylthiazol-2-yl)-2,5-diphenyltetrazolium bromide
PCNA: proliferating cell nuclear antigen
PI: propidium iodide
RT-qPCR: real-time polymerase chain reaction-quantitative polymerase chain reaction
SR: serine arginine amino acid residues
TBST: Tris buffered saline Tween
